# Abnormal Brain Network Interaction Associated With Positive Symptoms in Drug-Naive Patients With First-Episode Schizophrenia

**DOI:** 10.3389/fpsyt.2022.870709

**Published:** 2022-05-17

**Authors:** Liu Yuan, Xiaoqian Ma, David Li, Zongchang Li, Lijun Ouyang, Lejia Fan, Zihao Yang, Zhenmei Zhang, Chunwang Li, Ying He, Xiaogang Chen

**Affiliations:** ^1^Department of Psychiatry, National Clinical Research Center for Mental Disorders, The Second Xiangya Hospital of Central South University, Changsha, China; ^2^Mental Health Institute of Central South University, China National Technology Institute on Mental Disorders, Hunan Technology Institute of Psychiatry, Hunan Key Laboratory of Psychiatry and Mental Health, Changsha, China; ^3^Department of Radiology, Hunan Children’s Hospital, Changsha, China

**Keywords:** schizophrenia, positive symptoms, CPM, brain network, segregation

## Abstract

Positive symptoms are marked features of schizophrenia, and emerging evidence has suggested that abnormalities of the brain network underlying these symptoms may play a crucial role in the pathophysiology of the disease. We constructed two brain functional networks based on the positive and negative correlations between positive symptom scores and brain connectivity in drug-naive patients with first-episode schizophrenia (FES, *n* = 45) by using a machine-learning approach (connectome-based predictive modeling, CPM). The accuracy of the model was *r* = 0.47 (*p* = 0.002). The positively and negatively associated network strengths were then compared among FES subjects, individuals at genetic high risk (GHR, *n* = 41) for schizophrenia, and healthy controls (HCs, *n* = 48). The results indicated that the positively associated network contained more cross-subnetwork connections (96.02% of 176 edges), with a focus on the default-mode network (DMN)-salience network (SN) and the DMN-frontoparietal task control (FPT) network. The negatively associated network had fewer cross-subnetwork connections (71.79% of 117 edges) and focused on the sensory/somatomotor hand (SMH)-Cingulo opercular task control (COTC) network, the DMN, and the visual network with significantly decreased connectivity in the COTC-SMH network in FES (FES < GHR, *p* = 0.01; FES < HC, *p* = 0.01). Additionally, the connectivity strengths of the right supplementary motor area (SMA) (*p* < 0.001) and the right precentral gyrus (*p* < 0.0001) were reduced in FES. To the best of our knowledge, this is the first study to generate two brain networks associated with positive symptoms by utilizing CPM in FES. Abnormal segregation, interactions of brain subnetworks, and impaired SMA might lead to salience attribution abnormalities and, thus, as a result, induce positive symptoms in schizophrenia.

## Introduction

Positive symptoms are distinguishing characteristics of schizophrenia; a severe mental disorder with diverse symptoms. Among these positive symptoms, the most prominent include hallucinations, delusions, and disordered thinking and behavior. The hallucination-delusion syndrome can lead to serious impairment of social function in patients. Currently, the primary goal of antipsychotic drugs in the treatment of schizophrenia is to reduce the occurrences and severity of positive symptoms. Exploring the underlying mechanisms of positive symptoms can assist in expediting the search for the etiology and potential therapies for the disease.

The “disconnection” hypothesis in schizophrenia is widely accepted. Both the abnormal salience monitoring theory of schizophrenia (1, 2) and the triple network model of psychopathology (3) posit that the abnormal functional communication between the SN and the DMN, or among the DMN, or between the SN the central executive network (CEN) is the mechanism behind psychotic symptoms. A recent study conducted by using deep discriminant autoencoder networks revealed that the dysfunctional integration within and across the DMN, the SN, and the frontoparietal task control (FPT) network plays an important role in the “disconnectivity” model of schizophrenia (4). A lower integration of the DMN, the CEN, and the sensory/somatomotor hand (SMH) network is associated with the severity of positive symptoms (5). Reduced DMN and CEN activation is observed in delusional patients with schizophrenia (6). Prior studies have reported strong relationships between disrupted SN/DMN functional communication and positive symptoms (7). All this emerging evidence implicate the occurrence of mental symptoms is closely related to the communication between large-scale brain networks.

With the development of technology, machine learning (ML) has significantly impacted the field of neuroscience. Thus, we aim to explore brain network connections based on a whole-brain, ML approach. Connectome-based predictive modeling (CPM) is one such method used for developing predictive models of brain-behavior relationships from whole-brain functional connectivity, with cross-validation to improve statistical efficacy and generalizability (8). In previous studies, CPM demonstrated adequate performances in predictive fluid intelligence (9), sustained attention (10), and cocaine abstinence (11). As CPM is based on linear modeling and a purely data-driven protocol, it exhibits suitable interpretability and presents two brain networks related to positive symptoms.

This study included patients with FES as the subjects. As FES subjects had never been treated, unlike patients with chronic schizophrenia, connectivity abnormalities may be distinguished without the confounding effects of medications. CPM was utilized here to extract the most relevant features (connections) associated with positive symptoms in patients with FES across the brain and for the construction of two functional networks. The connections between different functional subnetworks were then observed by using the networks identified previously with CPM. Meanwhile, we chose subjects at genetic high risk (GHR) for schizophrenia and healthy controls (HCs) and compared their network connectivity with that of the FES group. Compared to HCs, GHR subjects were at a higher risk of developing schizophrenia (12) and showed cognitive deficits (13–15), but they did not suffer from severe psychiatric symptoms. As positive symptoms represent a pathological state, we explored the network alteration in the FES group by comparing it with asymptomatic people. We hypothesized that positive symptoms are associated with changes in brain connectivity, especially in connections between different functional subnetworks.

## Materials and Methods

### Participants

The study participants comprised 48 drug-naive patients with FES, 41 GHR subjects, and 50 HCs. The FES subjects were recruited from the Department of Psychiatry of the Second Xiangya Hospital of Central South University in China, while the GHR subjects and HCs were recruited from the local community. The FES subjects were diagnosed through a structured clinical interview according to the DSM-5 criteria. The GHR subjects had at least one first-degree relative who suffered from schizophrenia. All participants were drug-naive, 13–35 years old, right-handed, and Han Chinese. Subjects with a history of neurological or severe physical disease, substance abuse, or with an IQ of <70, which was determined by the WASI-IV (16), were excluded from the study. This study was approved by the ethics committee of the Second Xiangya Hospital of Central South University. Written informed consent was obtained from all subjects or their guardians.

### Positive and Negative Syndrome Scale

The Positive and Negative Syndrome Scale (PANSS) (17) was used to evaluate the psychiatric symptomatology of the FES subjects. The assessments were conducted by clinical psychiatrists with experience and expertise in PANSS assessment. The total score for positive symptoms of each FES subject was the sum of all items in the positive subscale.

### Image Acquisition

For each participant, magnetic resonance imaging (MRI) data were acquired by using a 3.0T magnetic resonance imager (Siemens, Skyra, Germany), equipped with a 16-channel array coil at Hunan Children’s Hospital, Changsha, China. During the scan, the participants were required to remain motionless and awake with their eyes closed. Foam pads and earplugs were provided to minimize head motion. Rest data was collected with single-shot full k-space echo-planar imaging (EPI) and the sequence parameters were as follows: TR/TE = 2,000/30 ms, slice number = 36, flip angle = 90°, field of view (FOV) = 256 mm^2^ × 256 mm^2^, slice thickness = 3.4 mm, and voxel size = 3.4 mm^3^ × 3.4 mm^3^ × 3.4 mm^3^. For each participant, one functional run contained 250 image volumes within a 508 s scanning time. For registration of functional images, a high-resolution structural image was acquired using a high-resolution sequence: TR = 2,530 ms, TE = 2.33 ms, flip angle = 7°, slice number = 192, FOV = 256 mm^2^ × 256 mm^2^, slice thickness = 1 mm, and voxel size = 1 mm^3^ × 1 mm^3^ × 1 mm^3^.

### Data Preprocessing

Resting-state functional magnetic resonance imaging (rs-fMRI) data preprocessing was performed by using Data Processing Assistant for rs-fMRI (running in MATLAB R2013b) (18). The data pre-processing sequence was as follows. The first 10 time points of each functional image were removed to facilitate equilibration of the magnetic field. Slice timing and realignment were performed, followed by the execution of within-subject co-registration of the T1 image to a functional image and segmentation into gray matter (GM), white matter (WM), and cerebrospinal fluid (CSF). To reduce the effects of non-neuronal fluctuations, including the CSF and WM signals and head motion profiles, nuisance covariate regression was performed by using Friston’s 24-parameter model (19) (six head motion parameters, six head motion parameters one-time point before, and 12 corresponding squared items). Individual data were transformed into a standardized Montreal Neurological Institute coordinates (MNI) space by applying the normalization parameters obtained from DARTEL, with a resampling voxel size of 3 mm × 3 mm × 3 mm. The generated images were then smoothed by using a 4 mm^3^ × 4 mm^3^ × 4 mm^3^ full-width at half-maximum (FWHM) Gaussian kernel with the linear trends removed. Finally, the MRI data were bandpass filtered (0.01–0.1 Hz) to reduce the effects of low-frequency drift and high-frequency physiological noises. To control the quality of the fMRI data, pictures for normalization from each participant were presented and scored during preprocessing. One HC with serious normalization problems (score < 3) was excluded. To control the head motion, the discarded subjects were defined as mean FD (Jenkinson) of >0.2 mm (20). As a result, three FES subjects and one HC were excluded. We additionally calculated the mean FD (Jenkinson) (21) and compared it among the three groups with results demonstrating no significant difference in FD (*f* = 0.359, *p* = 0.783). The fMRI data of each subject were then divided into 264 brain regions by utilizing the Power264 atlas (22). A correlation analysis was finally performed between each of the two brain regions by using Pearson correlation to obtain 264 × 264 matrices with Fisher z-transformation.

### Construction of Positive Symptom-Associated Network by Connectome-Based Predictive Modeling

In this study, CPM used the leave-one-out cross-validation approach, where in each iteration, one FES subject formed the test set and the remaining FES subjects formed the training set. First, a 264 × 264 correlation matrix was obtained for each subject in the training set with each number representing the strength of the connection between the two brain regions. The total positive symptom scores of the FES subjects were normally distributed. Pearson correlation was used to correlate each value in the correlation matrix with the total positive symptom score and to obtain the statistical *p*-value. By setting a threshold value of *p* = 0.01, the most positively and negatively relevant edges were selected for model-building in the training set. The strengths of all positively associated connections, as well those of the negative connections, were summed. The two total values were entered into a linear model with the total positive symptom score. The generated model was used to predict the positive symptom scores of individuals in the test set. After 45 iterations, the predicted score of each patient was obtained. The connections that survived in each iteration were aggregated to form a final and positively associated network and a negatively associated network. The predicted scores were correlated with the actual scores to measure the model’s accuracy (*r*-value). Statistical significance for the model accuracy was assessed by using 10,000 permutation tests. Positive symptom scores were randomly reassigned to different subjects. Then, new label assignments were input to build a new model and obtain a new correlation coefficient. This process was repeated 10,000 times to generate an empirical null distribution to assess the statistical significance of the true model’s accuracy. The statistical threshold was set at *p* < 0.05.

Threshold selection: As mentioned above, the selection of significantly related connections is important in CPM and a threshold value of *p* = 0.01 has been used in several previous studies which utilized CPM (9, 10). However, since no consensus has been reached on the threshold value of *p*, we chose a series of thresholds from *p* = 0.005 to 0.05 with increments of 0.005. Consequently, 10 models under different thresholds were obtained. Each of these was found to be effective after the permutation test and all of them had similar accuracies. For the following subsequent analysis, we chose the model with *p* = 0.01 because it was more stringent in the selection of connections and enabled better performance (*r* = 0.47, *p* = 0.002). The accuracies of the remaining models are presented in the Supplementary Material.

### Connection Strength Comparison

In all three groups (FES, GHR, and HC), the total strength of the final positively/negatively associated network was calculated by summing all connections. First, in each group, the total strength of the positively associated network was compared with that of the negatively associated network. Second, the total strength of both the positively and negatively associated networks was compared among the three groups. Multiple comparison correction was then performed with Bonferroni correction.

We calculated the connection strengths between the different subnetworks according to the main subnetworks involved. In the positively associated network, the strengths of connections between the DMN and the FPT network along with the DMN-SN connections were summed. In the negatively associated network, the connection strengths of the SMH-Cingulo opercular task control (COTC) network, the DMN, and the visual network were calculated. These connection strengths were compared among the three groups. Multiple comparison correction was performed with Bonferroni correction.

Furthermore, the strength of each node in the positively/negatively associated network was calculated by summing the weights of all edges connected to the node. The nodes were then compared among the three groups and corrected with FDR (*q* < 0.05). Age, gender, years of education, and head motion (mean FD) were treated as covariates.

### Statistical Analysis

Data analysis was performed by using SPSS (IBM SPSS Statistics for Macintosh, Version 23.0). Mean FD, demographic, and clinical variables of the three groups were compared by using analysis of variance (ANOVA) or chi-squared test. For *post hoc* comparisons, Fisher’s least significant difference procedure and Dunnett T3 correction (adjusted *p* < 0.05) were applied. The network strength was examined by using an *F*-test with Bonferroni correction. Group differences in node strength were identified by ANOVA with FDR correction. Age, gender, years of education, and head motion (23) were regarded as covariates in all comparisons among the groups. The statistical threshold was set as *p* < 0.05.

## Results

### Demographic and Clinical Characteristics

No significant differences were found in terms of gender (*p* = 0.671), age (*p* = 0.602), and mean FD (*p* = 0.57) among the three groups. HC had a higher educational level than the other groups (*p* = 0.006; Table 1).

**TABLE 1:** Demographic and clinical information.

	FES	GHR	HC	*P* Value	X^2/^F
Gender (male/female)	23/22	23/18	29/19	0.67	0.817
Age (year)	20.81 ± 5.66	19.95 ± 4.84	20.02 ± 4.67	0.602	0.509
Education (year)	11.91 ± 3.00	11.73 ± 3.23	13.64 ± 3.05	0.006*	5.268
Mean FD Jenkinson	0.068 ± 0.04	0.06 ± 0.04	0.06 ± 0.03	0.57	0.564
Total PANSS positive score	22.24 ± 6.86				

*The difference is significant with a p value of less than 0.05.

### Defined Positive and Negative Networks Related to Positive Symptoms of First-Episode Schizophrenia

**FIGURE 1:**
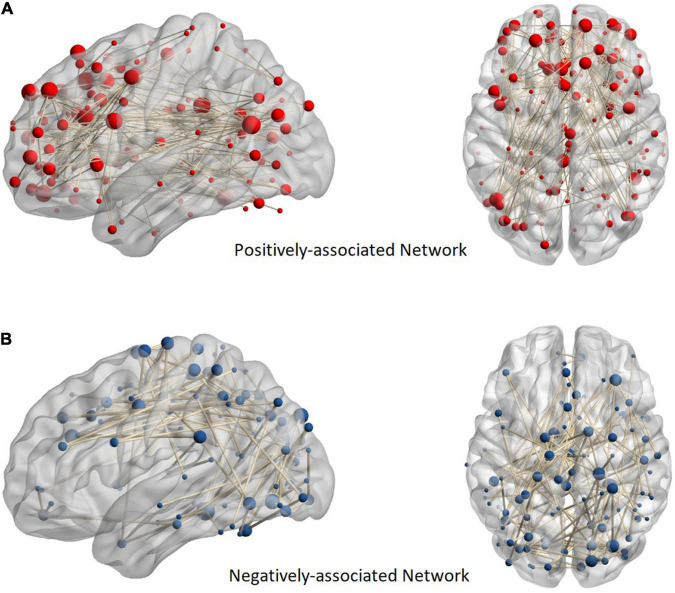
Anatomical distribution of positive and negative networks. Panel **(A)** shows a positively associated network and **(B)** shows a negatively associated network. The sphere in the figure represents the brain region. In addition, the more edges connected to the brain region, the larger the ball. The nodes of the positive network are focused on the frontal, temporal, and limbic systems, while those of the negative network are focused on the parietal and occipital lobes.

**FIGURE 2:**
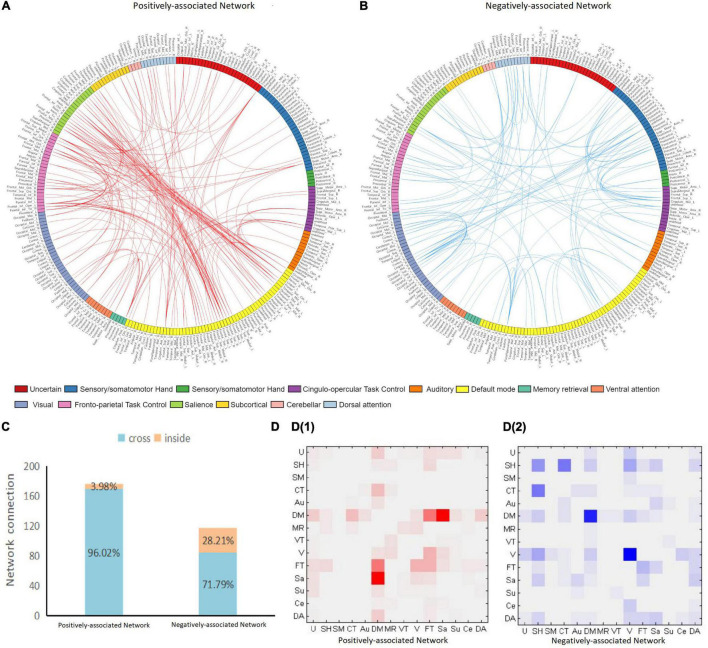
Positive and negative network connection. Panel **(A,B)** plots show the distribution of positively and negatively associated networks across the 14 subnetworks. The 264 brain regions on the map are marked with different colors, each representing a subnetwork. The anatomical label of each brain region corresponds to the AAL90 atlas by MNI coordinates. Red edges indicate a positively associated network and blue edges indicate a negatively associated network. Panel **(C)** shows the proportion of cross-and intra-subnetwork connections. Panel **(D)** shows the connection matrix in each subnetwork. D1 is the positively associated network, while D2 is the negatively associated network. The positively associated network connections are focused on the DMN-SN and DMN-FPT networks. The negatively associated networks are focused on COTC-SMH network, DMN, and the visual network. U, uncertain; SH, sensory/somatomotor hand; SM, sensory/somatomotor mouth; CT, Cingulo-opercular task control; Au, auditory; DM, default mode; MR, memory retrieval; VT, ventral attention; V, visual; FT, fronto-parietal task control; Sa, salience; Su, subcortical; Ce, cerebellar; DA, dorsal attention.

The positively and negatively associated networks in FES are shown in Figure 1. The obtained positively associated network indicated that a stronger network connection led to more serious positive symptoms, while the opposite was true for the negatively associated network. The positively associated network involved 127 brain regions with 176 edges, while the negatively associated network involved 108 brain regions with 117 edges. The positively associated connections focused on the frontal and temporal lobes and the limbic system, while the negatively associated connections focused on the frontal, parietal, and occipital lobes. Based on the Power264 atlas, which divides the whole brain into 14 subnetworks, cross-subnetwork connections were found to be dominant in positively associated networks (accounting for 96.02%). These connections were concentrated in the DMN-SN (27.27%) and the DMN-FPT network (13.64%). The negatively associated networks had fewer cross-subnetwork connections (71.79%) and were concentrated in the COTC-SMH network (11.11%), the visual network (11.11%), and DMN (9.40%) (Figure 2).

### Network Strength Alteration and Decreased Node Connectivity in First-Episode Schizophrenia

The total strength of the negatively associated network was significantly higher than that of the positively associated network in HCs (*p* < 0.001) and GHR subjects (*p* < 0.001). The difference between the two networks was not observed in the FES subjects. Although the negatively associated networks had fewer edges, they had higher connectivity strength in all individuals except for FES subjects.

**FIGURE 3:**
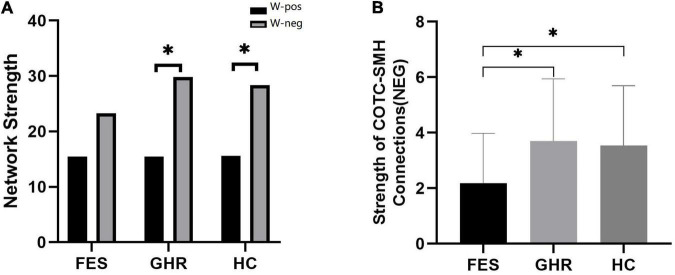
Comparison of positively and negatively associated network strengths and the decreased connectivity of the Cingulo opercular task control (COTC)-sensory/somatomotor hand (SMH0 network. **(A)** Negatively associated network strength was significantly higher than positively associated network strength in HCs (*p* < 0.001) and GHR (*p* < 0.001). **(B)** The strength of negatively associated connections in the COTC-SMH network in FES was significantly decreased as compared to GHR (*p* = 0.005) and HC (*p* = 0.015). *The difference was significant with a *p* value of less than 0.05 (after Bonferroni correction).

Neither positively nor negatively associated network strengths differed among the three groups. In the comparison of connectivity strength among different subnetworks, only connectivity strength between COTC and SMH in the negatively associated network exhibited a significant difference (FES < GHR, *p* = 0.01; FES < HC, *p* = 0.01; Table 2 and Figure 3).

**TABLE 2:** Comparison of network strength among the three groups [first episode schizophrenia (FES), genetic high-risk (GHR), and healthy controls (HCs)].

	FES (*n* = 45)	GHR (*n* = 41)	HC (*n* = 48)	FES vs HC		FES vs GHR		Between groups	
				*P*	T	*P*	T	*P*	F
Pos:DMN-SN	4.35 ± 6.99	4.01 ± 4.78	4.42 ± 4.45	–	–	–	–	0.93	0.068
Pos:DMN-FPT	2.33 ± 3.47	2.29 ± 2.23	2.33 ± 2.47	–	–	–	–	0.93	0.003
Neg:COTC-SMH	2.17 ± 1.8	3.7 ± 2.24	3.54 ± 2.16	0.01*	3.30	3.49	0.01*	0.01*	7.278
Neg:DMN-DMN	2.68 ± 1.68	2.74 ± 1.41	2.81 ± 1.31	–	–	–	–	0.92	0.085
Neg:Visual-Visual	4.74 ± 2.93	5.33 ± 3.59	5.18 ± 2.41	–	–	–	–	0.63	0.459
W-pos	15.50 ± 21.59	15.44 ± 12.50	15.56 ± 12.29	–	–	–	–	0.998	0.002
W-neg	23.25 ± 16.80	29.83 ± 17.64	28.37 ± 13.10	–	–	–	–	0.15	1.925
*P* value of W-_*pos*_ vs W-neg	0.60	0.000*	0.000*						
T value of W-_*pos*_ vs W-neg	-1.902	-4.262	-4.935						

*The difference was significant with a p value of less than 0.05 (after Bonferroni correction).

In the negatively associated network, the node strengths of P28 (Precentral_R, *p* < 0.0001), P21 (Precentral_R, *p* < 0.0001), and P54 (Supp_Motor_Area_R, *p* < 0.001) were significantly reduced in FES subjects compared to that in GHR subjects. No significant difference was observed in positively associated nodes.

## Discussion

Schizophrenia is considered a disorder of the brain’s network connection (24, 25). Recent studies have found both hyperconnectivity and hypoconnectivity in the brain network of individuals suffering from schizophrenia (26, 27). Similarly, in this study, two opposite networks associated with positive symptoms were defined in FES. The positively associated network had more cross-subnetwork connections (96.02%), which were concentrated in the DMN-SN and the DMN-FPT network. In comparison, the negatively associated network had fewer cross-subnetworks (71.79%), which were concentrated in the COTC-SMH network, the visual network, and the DMN. Although there were fewer edges, the total connectivity strength of the negatively associated network was significantly higher than that of the positively associated network in the HC and GHR groups. In contrast, this feature was not found in the FES group. Moreover, the connectivity strength between the COTC and the SMH networks and between the right Supplementary motor area (SMA) and the right precentral gyrus in the negatively associated network were significantly decreased in the FES group. Connectivity in the negatively associated network was decreased, while in the positively associated network, connectivity was increased with more subnetwork communications. This suggested that increased communication between brain subnetworks was related to higher positive symptom scores. In addition, network segregation was affected and interaction between the brain subnetworks was altered in the FES group.

The small-world property (28, 29) is an important feature of brain networks and has been well established. Excessive connectivity between subnetworks can cause considerable specialization in a community (30, 31), while increased resting-state system segregation is positively correlated with cognitive training improvements (32, 33). In contrast, brain dysfunction and reduced segregation of brain systems have been widely observed in many neuropsychiatric diseases, especially schizophrenia (34). These findings are similar to the results of the present study.

According to a previously proposed hypothesis, a resting-state organization represents an optimized state of metabolic energy demands and maintains segregation at rest to allow each functional system to respond rapidly and flexibly to the processing of tasks and goals (30). Thus, the imbalance of segregation and integration of brain subnetworks (especially the associated systems in positively associated networks) can lead to the disability of higher-order cognitive functions.

Among the mainly involved subnetworks in positively associated connections, The DMN, one of the primary subnetworks involved in positively associated connections, focuses on different aspects of self-referential processing (35). The FPT network, also known as the CEN (36), is essential for decision-making in the context of goal-directed behavior (37, 38), while the SN acts as a switch between the CEN and the DMN (3, 39–41). The DMN and the SN appear to have more of a negative correlation. However, in this study, the connections among the DMN, the SN, and the CEN were enhanced. Based on the deficits of the DMN, the SN, and the CEN in patients with schizophrenia (3, 7, 26, 42, 43), Menon (3) proposed the triple network model, suggesting that abnormalities in the engagement and disengagement of these three core networks play an important role in psychiatric disorders. An important aspect is the inappropriate salience assignment to external stimuli or internal events, leaving the cognitive system lacking in context-relevant engagement and disengagement. The symptoms in subjects at risk of psychosis are also associated with reality distortion (44). Hence, we considered that the abnormal interactions among the DMN, the SN, and the CEN might be related to inappropriate salience assignment and interfere with higher-level cognition with potential induction of psychiatric symptoms. A recent meta-analysis of schizophrenia (26) indicated hyper-connectivity between the affected network and the ventral attention network, implicating that the imbalanced communication between the salience processing network and other networks made it difficult for patients to distinguish between the internal and external worlds.

Another important mechanism of the triple network model is the aberrant bottom-up detection of salient events. In this study, reduced connectivity in the COTC-SMH network was found in the negatively associated network, indicating that positive symptoms are associated with low-level sensory signal changes in cognitive processing. The COTC network shares similar functions and anatomical locations with the SN (36, 45) and is in charge of initiating and maintaining task processing (46, 47). The decreased connections between COTC and the perception network might reflect the unusually detected signals of perception. The inability to accurately initiate and maintain the task process might cause abnormal attention allocation. This result is consistent with those obtained by Orliac, who discovered a pronounced effect of reduced functional connectivity in the visual, auditory, and cross-modal binding networks in patients with schizophrenia (48).

Another important result of this study was the reduced connectivity strength of the right SMA and the right precentral gyrus in the FES group. In patients with schizophrenia, the precentral gyrus is involved in auditory-verbal imagery (49), and its activity is associated with auditory verbal hallucinations (50). In psychopathological studies, SMA’s function is considered necessary for self-attribution (51–53). A study found that schizophrenia patients with hallucinations have low SMA activity in tasks requiring the generation and monitoring of the inner language (54). SMA activation in healthy individuals with non-clinical hallucinations is related to a lack of self-control of hallucinations (55). The impaired function of SMA can diminish a person’s capacity for voluntary action and mental imagery (49, 56, 57), leading to misinterpreted intentions or speech perceptions that form the foundations of hallucinations and illusions (52, 58).

Both the affected interactions between subnetworks and the decreased connectivity of SMA indicate that the inappropriate salience assignment is closely related to the positive symptoms in patients with schizophrenia. From a pharmacological perspective, midbrain dopamine receptors are the therapeutic target for positive symptoms (59). Abnormal dopamine regulation of the mesolimbic system in schizophrenia might lead to aberrant attribution of saliency and contribute to the emergence of positive symptoms (60–62). Patients with schizophrenia are highly attentive to irrelevant cues and are positively correlated with positive symptoms (63). Therefore, we suggest that abnormal subnetwork interactions and segregation can have cascading consequences on attention allocation and engagement of the cognitive system. Impaired SMA can lead to abnormal attribution, causing hallucinations.

A limitation of this study was that another independent set was not used to validate the CPM model. Therefore, the positively and negatively associated networks extracted in this study might not apply to all individuals with schizophrenia. On one hand, it was difficult to ensure consistency in PANSS assessment across studies. The derived brain network based on FES in this study might not be able to accurately predict the PANSS scores of subjects in other studies. On the other hand, in clinical practice, clinicians do not need to use CPM to assess the positive symptoms of schizophrenia. Given the strong interpretability of CPM, we sought to extract brain networks that were significantly associated with positive symptoms. After rigorous cross-validation and permutation tests, we concluded that the functional network extracted by CPM is significantly correlated with positive symptoms. Another limitation of this study was that the age of the FES group was between 13 and 35 years. Although age was treated as a covariate, it was difficult to remove any potential influence. In future studies, more subjects should be included to cover all age stages.

## Conclusion

In this study, we found that increased communication among different functional modules is related to higher positive symptoms scores. Abnormal interactions among the DMN, the SN, and the CEN along with decreased connectivity in the COTC-SMH network could be involved in the development of positive symptoms. The hypofunction of SMA might cause abnormal attributions of the internal language. These might interfere with the patient’s recognition of internal and external stimuli, contributing to the bias of thought. Thus, more in-depth neurobiological studies need to be conducted to explore the function and interactions of large-scale brain networks.

## Data Availability Statement

The original contributions presented in the study are included in the article/Supplementary Material, further inquiries can be directed to the corresponding authors.

## Ethics Statement

The studies involving human participants were reviewed and approved by Ethics Committee of the Second Xiangya Hospital of Central South University. Written informed consent to participate in this study was provided by the participants’ legal guardian/next of kin.

## Author Contributions

All authors have contributed to and have approved of the final manuscript.

## Conflict of Interest

The authors declare that the research was conducted in the absence of any commercial or financial relationships that could be construed as a potential conflict of interest.

## Publisher’s Note

All claims expressed in this article are solely those of the authors and do not necessarily represent those of their affiliated organizations, or those of the publisher, the editors and the reviewers. Any product that may be evaluated in this article, or claim that may be made by its manufacturer, is not guaranteed or endorsed by the publisher.

## References

[1] Palaniyappan L Liddle PF. Does the salience network play a cardinal role in psychosis? An emerging hypothesis of insular dysfunction. *J Psychiatry Neurosci.* (2012) 37:17–27. 10.1503/jpn.100176 21693094 PMC3244495

[2] Menon V. Salience network. In: Toga AW editor. *Brain Mapping: An Encyclopedic Reference.* Cambridge, MA: Academic Press (2015). p. 597–611. 10.1016/B978-0-12-397025-1.00052-X

[3] Menon V. Large-scale brain networks and psychopathology: a unifying triple network model. *Trends Cogn Sci.* (2011) 15:483–506. 10.1016/j.tics.2011.08.003 21908230

[4] Zeng LL Wang H Hu P Yang B Pu W Shen H Multi-site diagnostic classification of schizophrenia using discriminant deep learning with functional connectivity MRI. *Ebiomedicine.* (2018) 30:74–85. 10.1016/j.ebiom.2018.03.017 29622496 PMC5952341

[5] Lee WH Doucet GE Leibu E Frangou S. Resting-state network connectivity and metastability predict clinical symptoms in schizophrenia. *Schizophr Res.* (2018) 201:208–16. 10.1016/j.schres.2018.04.029 29709491 PMC6317903

[6] Lavigne KM Menon M Woodward TS. Functional brain networks underlying evidence integration and delusions in schizophrenia. *Schizophr Bull.* (2020) 46:175–83. 10.1093/schbul/sbz032 31050762 PMC6942156

[7] Hare SM Ford JM Mathalon DH Damaraju E Bustillo J Belger A Salience-Default mode functional network connectivity linked to positive and negative symptoms of schizophrenia. *Schizophr Bull.* (2019) 45:892–901. 10.1093/schbul/sby112 30169884 PMC6581131

[8] Shen X Finn ES Scheinost D Rosenberg MD Chun MM Papademetris X Using connectome-based predictive modeling to predict individual behavior from brain connectivity. *Nat Protoc.* (2017) 12:506–18. 10.1038/nprot.2016.178 28182017 PMC5526681

[9] Finn ES Shen X Scheinost D Rosenberg MD Huang J Chun MM Functional connectome fingerprinting: identifying individuals using patterns of brain connectivity. *Nat Neurosci.* (2015) 18:1664–71. 10.1038/nn.4135 26457551 PMC5008686

[10] Rosenberg MD Finn ES Scheinost D Papademetris X Shen X Constable RT A neuromarker of sustained attention from whole-brain functional connectivity. *Nat Neurosci.* (2016) 19:165–71. 10.1038/nn.4179 26595653 PMC4696892

[11] Yip SW Scheinost D Potenza MN Carroll KM. Connectome-based prediction of cocaine abstinence. *Am J Psychiatry.* (2019) 176:156–64. 10.1176/appi.ajp.2018.17101147 30606049 PMC6481181

[12] Gottesman II Gould TD. The endophenotype concept in psychiatry: etymology and strategic intentions. *Am J Psychiatry.* (2003) 160:636–45. 10.1176/appi.ajp.160.4.636 12668349

[13] Spilka MJ Goghari VM. Similar patterns of brain activation abnormalities during emotional and non-emotional judgments of faces in a schizophrenia family study. *Neuropsychologia.* (2017) 96:164–74. 10.1016/j.neuropsychologia.2017.01.014 28093278

[14] Tang Y Chen K Zhou Y Liu J Wang Y Driesen N Neural activity changes in unaffected children of patients with schizophrenia: a resting-state fMRI study. *Schizophr Res.* (2015) 168:360–5. 10.1016/j.schres.2015.07.025 26232869

[15] Villarreal MF Drucaroff LJ Goldschmidt MG de Achaval D Costanzo EY Castro MN Pattern of brain activation during social cognitive tasks is related to social competence in siblings discordant for schizophrenia. *J Psychiatr Res.* (2014) 56:120–9. 10.1016/j.jpsychires.2014.05.011 24927685

[16] Wechsler D. *Wechsler Adult Intelligence Scale.* 4th ed. San Antonio, TX: Pearson Assessment (2008).

[17] Kay SR Fiszbein A Opler LA. The positive and negative syndrome scale (PANSS) for schizophrenia. *Schizophr Bull.* (1987) 13:261–76. 10.1093/schbul/13.2.261 3616518

[18] Chao-Gan Y Yu-Feng Z. DPARSF: a MATLAB toolbox for “pipeline” data analysis of resting-state fMRI. *Front Syst Neurosci.* (2010) 4:13. 10.3389/fnsys.2010.00013 20577591 PMC2889691

[19] Friston KJ Williams S Howard R Frackowiak RS Turner R. Movement-related effects in fMRI time-series. *Magn Reson Med.* (1996) 35:346–55. 10.1002/mrm.1910350312 8699946

[20] Yan CG Craddock RC Zuo XN Zang YF Milham MP. Standardizing the intrinsic brain: towards robust measurement of inter-individual variation in 1000 functional connectomes. *Neuroimage.* (2013) 80:246–62. 10.1016/j.neuroimage.2013.04.081 23631983 PMC4074397

[21] Jenkinson M Bannister P Brady M Smith S. Improved optimization for the robust and accurate linear registration and motion correction of brain images. *Neuroimage.* (2002) 17:825–41. 10.1006/nimg.2002.1132 12377157

[22] Power JD Cohen AL Nelson SM Wig GS Barnes KA Church JA Functional network organization of the human brain. *Neuron.* (2011) 72:665–78. 10.1016/j.neuron.2011.09.006 22099467 PMC3222858

[23] Zeng LL Wang D Fox MD Sabuncu M Hu D Ge M Neurobiological basis of head motion in brain imaging. *Proc Natl Acad Sci U S A.* (2014) 111:6058–62. 10.1073/pnas.1317424111 24711399 PMC4000812

[24] van den Heuvel MP Sporns O Collin G Scheewe T Mandl RCW Cahn W Abnormal rich club organization and functional brain dynamics in schizophrenia. *JAMA Psychiatry.* (2013) 70:783–92. 10.1001/jamapsychiatry.2013.1328 23739835

[25] van den Heuvel MP Fornito A. Brain networks in schizophrenia. *Neuropsychol Rev.* (2014) 24:32–48. 10.1007/s11065-014-9248-7 24500505

[26] Dong D Wang Y Chang X Luo C Yao D. Dysfunction of large-scale brain networks in schizophrenia: a meta-analysis of resting-state functional connectivity. *Schizophr Bull.* (2018) 44:168–81. 10.1093/schbul/sbx034 28338943 PMC5767956

[27] Lottman KK Gawne TJ Kraguljac NV Killen JF Reid MA Lahti AC. Examining resting-state functional connectivity in first-episode schizophrenia with 7T fMRI and MEG. *Neuroimage Clin.* (2019) 24:101959. 10.1016/j.nicl.2019.101959 31377556 PMC6677917

[28] Watts DJ Strogatz SH. Collective dynamics of ‘small-world’ networks. *Nature.* (1998) 393:440–2. 10.1038/30918 9623998

[29] Bullmore E Sporns O. Complex brain networks: graph theoretical analysis of structural and functional systems. *Nat Rev Neurosci.* (2009) 10:186–98. 10.1038/nrn2575 19190637

[30] Wig GS. Segregated systems of human brain networks. *Trends Cogn Sci.* (2017) 21:981–96. 10.1016/j.tics.2017.09.006 29100737

[31] Chan MY Park DC Savalia NK Petersen SE Wig GS. Decreased segregation of brain systems across the healthy adult lifespan. *Proc Natl Acad Sci U S A.* (2014) 111:E4997–5006. 10.1073/pnas.1415122111 25368199 PMC4246293

[32] Duncan ES Small SL. Increased modularity of resting state networks supports improved narrative production in aphasia recovery. *Brain Connect.* (2016) 6:524–9. 10.1089/brain.2016.0437 27345466 PMC5084363

[33] Gallen CL Baniqued PL Chapman SB Aslan S Keebler M Didehbani N Modular brain network organization predicts response to cognitive training in older adults. *PLoS One.* (2016) 11:e169015. 10.1371/journal.pone.0169015 28006029 PMC5179237

[34] Yang GJ Murray JD Wang XJ Glahn DC Pearlson GD Repovs G Functional hierarchy underlies preferential connectivity disturbances in schizophrenia. *Proc Natl Acad Sci U S A.* (2016) 113:E219–28. 10.1073/pnas.1508436113 26699491 PMC4720350

[35] Greicius MD Krasnow B Reiss AL Menon V. Functional connectivity in the resting brain: a network analysis of the default mode hypothesis. *Proc Natl Acad Sci U S A.* (2003) 100:253–8. 10.1073/pnas.0135058100 12506194 PMC140943

[36] Miyata J. Toward integrated understanding of salience in psychosis. *Neurobiol Dis.* (2019) 131:104414. 10.1016/j.nbd.2019.03.002 30849509

[37] Muller NG Knight RT. The functional neuroanatomy of working memory: contributions of human brain lesion studies. *Neuroscience.* (2006) 139:51–8. 10.1016/j.neuroscience.2005.09.018 16352402

[38] Petrides M. Lateral prefrontal cortex: architectonic and functional organization. *Philos Trans R Soc Lond B Biol Sci.* (2005) 360:781–95. 10.1098/rstb.2005.1631 15937012 PMC1569489

[39] Sridharan D Levitin DJ Menon V. A critical role for the right fronto-insular cortex in switching between central-executive and default-mode networks. *Proc Natl Acad Sci U S A.* (2008) 105:12569–74. 10.1073/pnas.0800005105 18723676 PMC2527952

[40] Chen AC Oathes DJ Chang C Bradley T Zhou ZW Williams LM Causal interactions between fronto-parietal central executive and default-mode networks in humans. *Proc Natl Acad Sci U S A.* (2013) 110:19944–9. 10.1073/pnas.1311772110 24248372 PMC3856839

[41] Seeley WW Menon V Schatzberg AF Keller J Glover GH Kenna H Dissociable intrinsic connectivity networks for salience processing and executive control. *J Neurosci.* (2007) 27:2349–56. 10.1523/JNEUROSCI.5587-06.2007 17329432 PMC2680293

[42] O’Neill A Mechelli A Bhattacharyya S. Dysconnectivity of large-scale functional networks in early psychosis: a meta-analysis. *Schizophr Bull.* (2019) 45:579–90. 10.1093/schbul/sby094 29982729 PMC6483589

[43] Broyd SJ Demanuele C Debener S Helps SK James CJ Sonuga-Barke EJ. Default-mode brain dysfunction in mental disorders: a systematic review. *Neurosci Biobehav Rev.* (2009) 33:279–96. 10.1016/j.neubiorev.2008.09.002 18824195

[44] Wotruba D Michels L Buechler R Metzler S Theodoridou A Gerstenberg M Aberrant coupling within and across the default mode, task-positive, and salience network in subjects at risk for psychosis. *Schizophr Bull.* (2014) 40:1095–104. 10.1093/schbul/sbt161 24243441 PMC4133671

[45] Seeley WW. The salience network: a neural system for perceiving and responding to homeostatic demands. *J Neurosci.* (2019) 39:9878–82. 10.1523/JNEUROSCI.1138-17.2019 31676604 PMC6978945

[46] Dosenbach NU Fair DA Cohen AL Schlaggar BL Petersen SE. A dual-networks architecture of top-down control. *Trends Cogn Sci.* (2008) 12:99–105. 10.1016/j.tics.2008.01.001 18262825 PMC3632449

[47] Sadaghiani S Scheeringa R Lehongre K Morillon B Giraud AL Kleinschmidt A. Intrinsic connectivity networks, alpha oscillations, and tonic alertness: a simultaneous electroencephalography/functional magnetic resonance imaging study. *J Neurosci.* (2010) 30:10243–50. 10.1523/JNEUROSCI.1004-10.2010 20668207 PMC6633365

[48] Orliac F Delamillieure P Delcroix N Naveau M Brazo P Razafimandimby A Network modeling of resting state connectivity points towards the bottom up theories of schizophrenia. *Psychiatry Res Neuroimaging.* (2017) 266:19–26. 10.1016/j.pscychresns.2017.04.003 28554165

[49] Qiu L Yan H Zhu R Yan J Yuan H Han Y Correlations between exploratory eye movement, hallucination, and cortical gray matter volume in people with schizophrenia. *BMC Psychiatry.* (2018) 18:226. 10.1186/s12888-018-1806-8 30005610 PMC6045825

[50] van Lutterveld R Diederen KM Koops S Begemann MJ Sommer IE. The influence of stimulus detection on activation patterns during auditory hallucinations. *Schizophr Res.* (2013) 145:27–32. 10.1016/j.schres.2013.01.004 23375942

[51] Wolpert DM Ghahramani Z Jordan MI. An internal model for sensorimotor integration. *Science.* (1995) 269:1880–2. 10.1126/science.7569931 7569931

[52] Frith C. The neural basis of hallucinations and delusions. *C R Biol.* (2005) 328:169–75. 10.1016/j.crvi.2004.10.012 15771003

[53] Rotarska-Jagiela A van de Ven V Oertel-Knochel V Uhlhaas PJ Vogeley K Linden DE. Resting-state functional network correlates of psychotic symptoms in schizophrenia. *Schizophr Res.* (2010) 117:21–30. 10.1016/j.schres.2010.01.001 20097544

[54] Stephane M Hagen MC Lee JT Uecker J Pardo PJ Kuskowski MA About the mechanisms of auditory verbal hallucinations: a positron emission tomographic study. *J Psychiatry Neurosci.* (2006) 31:396–405. 17136217 PMC1635803

[55] Linden DE Thornton K Kuswanto CN Johnston SJ van de Ven V Jackson MC. The brain’s voices: comparing nonclinical auditory hallucinations and imagery. *Cereb Cortex.* (2011) 21:330–7. 10.1093/cercor/bhq097 20530219

[56] Raij TT Riekki TJ. Poor supplementary motor area activation differentiates auditory verbal hallucination from imagining the hallucination. *Neuroimage Clin.* (2012) 1:75–80. 10.1016/j.nicl.2012.09.007 24179739 PMC3757718

[57] Haggard P. Human volition: towards a neuroscience of will. *Nat Rev Neurosci.* (2008) 9:934–46. 10.1038/nrn2497 19020512

[58] Ford JM Mathalon DH Heinks T Kalba S Faustman WO Roth WT. Neurophysiological evidence of corollary discharge dysfunction in schizophrenia. *Am J Psychiatry.* (2001) 158:2069–71. 10.1176/appi.ajp.158.12.2069 11729029

[59] Fusar-Poli P Meyer-Lindenberg A. Striatal presynaptic dopamine in schizophrenia, part II: meta-analysis of [(18)F/(11)C]-DOPA PET studies. *Schizophr Bull.* (2013) 39:33–42. 10.1093/schbul/sbr180 22282454 PMC3523905

[60] Kapur S. Psychosis as a state of aberrant salience: a framework linking biology, phenomenology, and pharmacology in schizophrenia. *Am J Psychiatry.* (2003) 160:13–23. 10.1176/appi.ajp.160.1.13 12505794

[61] Heinz A. Dopaminergic dysfunction in alcoholism and schizophrenia–psychopathological and behavioral correlates. *Eur Psychiatry.* (2002) 17:9–16. 10.1016/s0924-9338(02)00628-4 11918987

[62] Heinz A Schlagenhauf F. Dopaminergic dysfunction in schizophrenia: salience attribution revisited. *Schizophr Bull.* (2010) 36:472–85. 10.1093/schbul/sbq031 20453041 PMC2879696

[63] Morris R Griffiths O Le Pelley ME Weickert TW. Attention to irrelevant cues is related to positive symptoms in schizophrenia. *Schizophr Bull.* (2013) 39:575–82. 10.1093/schbul/sbr192 22267535 PMC3627774

