# Supplementary material for: Abnormal Brain Network Interaction Associated With Positive Symptoms in Drug-Naive Patients With First-Episode Schizophrenia
Source: Front Psychiatry. 2022 May 17;13:870709. doi: 10.3389/fpsyt.2022.870709 (PMC9152123; doi:10.3389/fpsyt.2022.870709)
Supplement: Supplementary file 1 [file Table_1.docx]

Supplementary Material

**Connectome-based predictive modeling**

The CPM approach was based on functional brain connectivity, resulting in a model that predicted behavioral scores. In this study, we chose a series of thresholds starting from P=0.005 and ending at 0.05 with incremental steps by 0.005. A total of 10 models with different thresholds and their accuracy were obtained. See Table 1.

Table 1. The accuracy metrics for 10 CPM models.

| P threshold of CPM | 0.005 | 0.01 | 0.015 | 0.02 | 0.025 | 0.03 | 0.035 | 0.04 | 0.045 | 0.05 |
| --- | --- | --- | --- | --- | --- | --- | --- | --- | --- | --- |
| R of model | 0.42 | 0.47 | 0.48 | 0.49 | 0.50 | 0.51 | 0.51 | 0.51 | 0.50 | 0.49 |
| P of model | 0.004 | 0.001 | 0.001 | 0.0006 | 0.0004 | 0.0003 | 0.0003 | 0.0004 | 0.0005 | 0.0006 |
| P after 10000 permutation | 0.014 | 0.002 | 0.002 | 0.001 | 0.001 | 0.0004 | 0.0003 | 0.0004 | 0.0006 | 0.0007 |
